# CircRNA CDR1as promotes hepatoblastoma proliferation and stemness by acting as a miR-7-5p sponge to upregulate KLF4 expression

**DOI:** 10.18632/aging.103748

**Published:** 2020-10-14

**Authors:** Luping Chen, Juanyi Shi, Yaohao Wu, Ronglin Qiu, Lexiang Zeng, Lei Lou, Jianhang Su, Minyi Liao, Xiaogeng Deng

**Affiliations:** 1 Department of Pediatric Surgery, Sun Yat-Sen Memorial Hospital, Sun Yat-Sen University, Guangzhou 510120, China; 2 Guangdong Provincial Key Laboratory of Malignant Tumor Epigenetics and Gene Regulation, Sun Yat-Sen Memorial Hospital, Sun Yat-Sen University, Guangzhou 510120, China

**Keywords:** hepatoblastoma, stemness, circRNA, CDR1as, miR-7-5p

## Abstract

Hepatoblastoma (HB) is a malignant embryonal tumor of the liver that consists of heterogenous populations of stem/progenitor cells. Although circular RNAs (circRNAs) play an essential role in tumor development, the effects of circRNA on the proliferation of HB cells, especially cancer stem cells (CSCs), remain unclear. We found that the circRNA, CDR1as, was highly expressed in CSC-enriched populations of HB cell lines. Results from flow cytometric and sphere-forming assays revealed that CDR1as knockdown in HB cell lines decreased the proportion of stem cells. The Cell Counting Kit-8 (CCK-8) assay, colony formation experiments, and EdU assay revealed that CDR1as knockdown in HB cell lines decreased cell growth and the colony-forming abilities. Biotin-coupled probe pull-down assays and biotin-coupled microRNA capture were conducted to evaluate the interaction between CDR1as and miR-7-5p. Dual-luciferase reporter assays demonstrated that Kruppel-like factor 4 (KLF4), expression of which is highly correlated with cancer stemness, was a target of miR-7-5p. Overall, the knockdown of CDR1as significantly inhibited the proliferation and stemness of HB cells by reducing the sponge activity on miR-7-5p and subsequently suppressing the interaction between miR-7-5p and KLF4. Results from this study suggest that CDR1as is an oncogene that effects the proliferation and stemness of HBs.

## INTRODUCTION

Cancers are a major cause of child death worldwide. Data from the World Health Organization (WHO) revealed that 397,000 children under the age of 15 developed cancer globally in 2015, and 43% of those cases went undiagnosed [1]. Due to barriers that limit the access to and referrals in the health system, the diagnosis of childhood cancer is inadequate, and nearly 50% of the global cases of childhood cancer have not been diagnosed nor treated. Since childhood cancer is such a serious condition, more research is needed to develop effective treatments.

There are multiple differences between childhood cancers and adult cancers. For example, adult cancers often involve a variety of genetic changes that drive cancer progress together, while childhood cancers are usually defined by a single driving gene [2]. Gröbner et al. [3] found that nearly 50% of primary childhood cancers harbor a targetable gene; therefore, it is necessary to study the mechanisms underlying the genetic changes that lead to cancer in children in order to improve the diagnosis of these cancers and to identify more effective gene therapies.

Hepatoblastoma (HB) is the most common primary malignant hepatic tumor in infants and children, and it predominantly occurs in the first two years of life [4, 5]. HB tumors consist of heterogenous populations of stem/progenitor cells [6–8]. Stemness provides cells with a strong driving force for uncontrolled growth and survival, which may also be associated with metastasis, drug resistance, and tumor recurrence. Cancer stem cells (CSCs) possess a high level of plasticity and may undergo undesired genetic, phenotypic, and epigenetic changes in response to the tumor microenvironment that increase their resistance to therapies and their ability to metastasize to other organs. However, the molecular and cellular bases of HB and the mechanisms underlying CSC induction are not fully understood.

Circular RNAs (circRNAs) are a novel class of RNA transcripts that are widely expressed in the mammalian genome. The 5′ and 3′ ends of circRNAs are covalently linked to form a closed circular structure. CircRNAs are usually more stable than their host genes, linear mRNAs, and they may also play a role in the development of some diseases, including hepatocellular carcinoma, renal cell carcinoma, and ovarian cancer [9–11]. As a newly recognized kind of functional RNA, circRNAs are also involved in the self-renewal of CSCs. Specifically, CircGprc5a, which is upregulated in bladder cancer and bladder CSCs, can be translated into a new short peptide that binds to Gprc5a and increases its function to drive the self-renewal of bladder CSCs [12]. Additionally, circ-ITCH functions as a sponge for miR-214, and miR-214 promotes the self-renewal and stemness of CSCs by repressing the expression of the Wnt-regulatory protein, CTNNBIP1 [13]. Results from these studies suggest that circRNAs play important roles in regulating the self-renewal of CSCs. However, studies have not evaluated the relationship between circRNA and CSCs in HB.

Kruppel-like factor 4 (KLF4) is a member of the Spl/Kruppel-like zinc-finger-containing transcription factors that plays a critical role in regulating a diverse array of cellular processes, including proliferation, differentiation, apoptosis, cellular migration, embryonic development, and maintenance of tissue homeostasis [14]. Additionally, KLF4 bi-directionally regulates genes that are involved in cell cycle regulation and epithelium differentiation [15]; functions as a tumor suppressor in malignant tumors, such as colorectal cancer and pancreatic ductal carcinoma [16, 17]; and functions as an oncogene in multiple cancers, including osteosarcoma, melanoma, and bladder cancer [18–20]. Further, KLF4 is one of the key factors that is used in induced pluripotent stem cell (iPSC) technology [21]; therefore, it may also be involved in the regulation of cancer stemness. Recent studies have indicated that KLF4 was required for the maintenance of cancer stem cells in osteosarcoma, breast cancer, and prostate cancer [18, 22, 23]. Although the mechanism(s) underlying these discrepancies remain unclear, evidence suggests that KLF4 plays an important role in cancer and highlights the need for further characterization of the specific role of KLF4 in CSCs.

The antisense cerebellar degenerative-related protein-1 (CDR1as) is the most widely investigated circRNA, and it acts a sponge for several microRNAs, such as miR-7, miR-1270, microRNA-135a, and miR-876-5p [24–27]. CDR1as also plays central roles in biological and pathological processes, including cell proliferation, metastasis, apoptosis, and chemosensitivity [24–27]. However, the role of CDR1as in HB has not been reported. Although HBs consist of heterogenous populations of stem/progenitor cells [6–8], the role of CDR1as in the development of cancer stem cells in HBs remains unknown. Therefore, we examined whether CDR1as affects the proliferation and self-renewal of CSCs in HB cell lines and explored the potential miRNA and downstream target genes.

## RESULTS

### Characteristics and expressions of circRNA CDR1as

**Figure 1:**
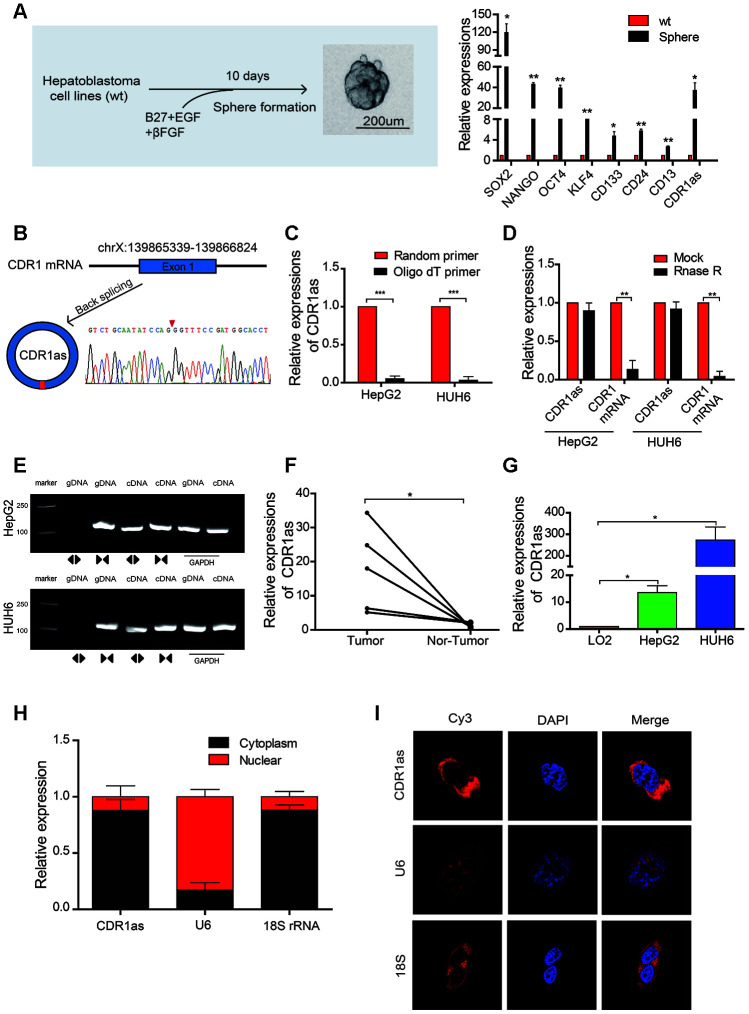
**The identification, characteristics, and expression of CDR1as in HB cell lines and HB.** (**A**) Schematic illustration of a forming HB sphere and qRT-PCR analysis for cancer stem cell (CSC) markers; (**B**) The formation of CDR1as. CDR1as is derived from back-spliced exon 1 of genomic CDR1. The existence of CDR1as was confirmed by Sanger sequencing. The red arrow represents the back-splice junction of CDR1as; (**C**) qRT-PCR analysis of CDR1as: reverse transcription products using random primers or oligo dT primers; (**D**) The expression of CDR1as and CDR1 mRNA was measured by qRT-PCR in HepG2 and HUH6 cells that were treated with or without RNase R; (**E**) The PCR products of CDR1as and linear CDR1 were evaluated by gel electrophoresis. Divergent primers amplified CDR1as in cDNA but not genomic DNA (gDNA). Convergent primers amplified linear CDR1 in both cDNA and gDNA. GAPDH was used as a linear control; (**F**) qRT-PCR analysis of CDR1as in 5 paired HB and adjacent noncancerous tissues; (**G**) The expression of CDR1as in HB cell lines was detected by qRT-PCR; (**H**) CDR1as was mainly located in the cytoplasm as confirmed by the nuclear mass separation assay in HepG2 cells; (**I**) Fluorescence in situ hybridization (FISH) confirmed that CDR1as was predominantly located in the cytoplasm. Nuclei were stained with DAPI. U6, 18S, and CDR1as were labeled with Cy3. Scale bar, 200 μm. Data are presented as the mean ± SEM of three experiments. *P < 0.05,**P < 0.01,***P < 0.001 (Student’s t-test).

We initially generated a CSCs-enriched population of spheres from HB cell lines and found that the expression of CSCs and liver CSCs markers, such as SOX2, OCT4, and CD133 [28, 29], was significantly higher in the CSCs-enriched spheres than that in the HB cell lines. Additionally, the expression of CDR1as was also significantly increased in the spheres as compared with that in the HB cells lines (Figure 1A). The circRNA, CDR1as (hsa_circ_0001946), was generated from the CDR1 gene and consisted of the head-to-toe splicing of exon 1. The head-to-toe splicing was confirmed in the CDR1as RT-PCR product and the size of the product was verified by Sanger sequencing (Figure 1B). Since circRNAs do not have a 3′ polyadenylated tail, they were not detected when oligo dT primers were used. The existence of CDR1as was detected in the reverse transcription products using random primers or oligo dT primers. These results indicated that CDR1as was almost undetectable when oligo dT primers were used (Figure 1C). The stability of circRNA is greater than the that of linear RNA because of the head-to-toe splicing. Although the CDR1 gene has only one exon, we designed convergent primers to amplify CDR1 mRNA and divergent primers to amplify CDR1as, because the circRNAs were not detected when oligo dT primers were used; the linear mRNA was not affected. Results from the RNAse assay demonstrated that the expression of CDR1as was not decreased, though the linear CDR1 was degraded after the digested with RNAse (Figure 1D). Using cDNA and genomic DNA (gDNA) from the HB cell lines as templates, we discovered that CDR1as was amplified in cDNA by divergent primers; however, no amplification products were observed in gDNA (Figure 1E). In addition, CDR1as expression was upregulated in HB tissues as compared with that in adjacent normal tissues (Figure 1F). Results from the qRT-PCR analysis also showed that the level of CDR1as was higher in the HB cell lines than that in the normal hepatocytes (Figure 1G). Furthermore, we conducted a qRT-PCR analysis and fluorescence in situ hybridization **(**FISH) assay to identify the cellular localization of CDR1as and found that CDR1as was mainly localized and distributed in the cytoplasm (Figure 1H–1I). These results demonstrated that CDR1as was upregulated in HB and HB CSCs and was predominantly localized in the cytoplasm.

### CDR1as knockdown inhibits the proliferation and stemness of HB cells *in vitro*

**Figure 2:**
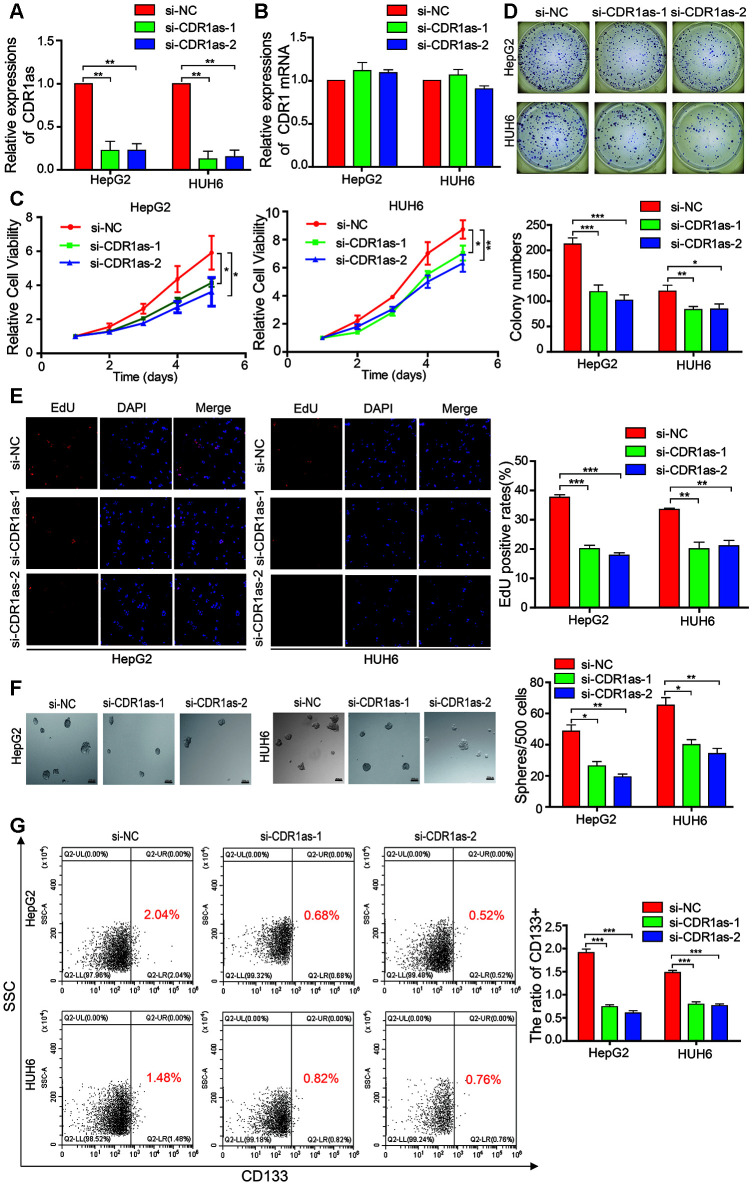
**CDR1as affects the proliferation and stemness of HB cells.** (**A**) qRT-PCR analysis of CDR1as in HB cells treated with siRNAs; (**B**) qRT-PCR analysis of CDR1 mRNA in HB cells treated with siRNAs; (**C**–**E**) Cell proliferation was assessed using the Cell Counting Kit-8 (CCK-8), colony formation assay, and EdU assay after knocking down CDR1as in HepG2 and HUH6 cells; (**F**) The stemness of the CSCs was assessed by the sphere-forming assay after knocking down CDR1as in HepG2 and HUH6 cells; (**G**) The percentage of HB cells expressing CD133 after knockdown of CDR1as was determined by flow cytometry. Scale bar, 200 μm. Data are presented as the mean ± SEM of three experiments. *P < 0.05,**P < 0.01,***P < 0.001 (Student’s t-test).

To test whether CDR1as affected the biological behavior of HB cells, we designed two siRNAs that targeted the back-splice region of CDR1as. Results from qRT-PCR revealed that CDR1as was successfully knocked down and CDR1 mRNA was not affected after the si-CDR1as was transfected into the HepG2 and HUH6 cells (Figure 2A, 2B). The Cell Counting Kit-8 (CCK-8) assay, colony formation experiments, and EdU assay revealed that cell growth and the colony-forming abilities of the HepG2 and HUH6 cells were decreased after CDR1as knockdown as compared with those of the control cells (Figure 2C–2E). The sphere-forming assay revealed that the sphere-forming capacity in the HB stem cells was decreased after CDR1as knockdown as compared with that in the control cells (Figure 2F). CD133 is a stem cell marker that is highly recognized in HB stem cells [30–32]. In the current study, CD133 expression was analyzed by flow cytometry in order to evaluate the proportion of CD133+ cells in the HB cell lines. Results from the flow cytometric analysis revealed that the proportion of CD133+ cells was significantly decreased in the knockdown CDR1as cells as compared with that in the control cells (Figure 2G). These findings suggested that CDR1as played a fundamental role in HB cell proliferation and stemness.

### CDR1as sponges miR-7-5p in HB cells

**Figure 3:**
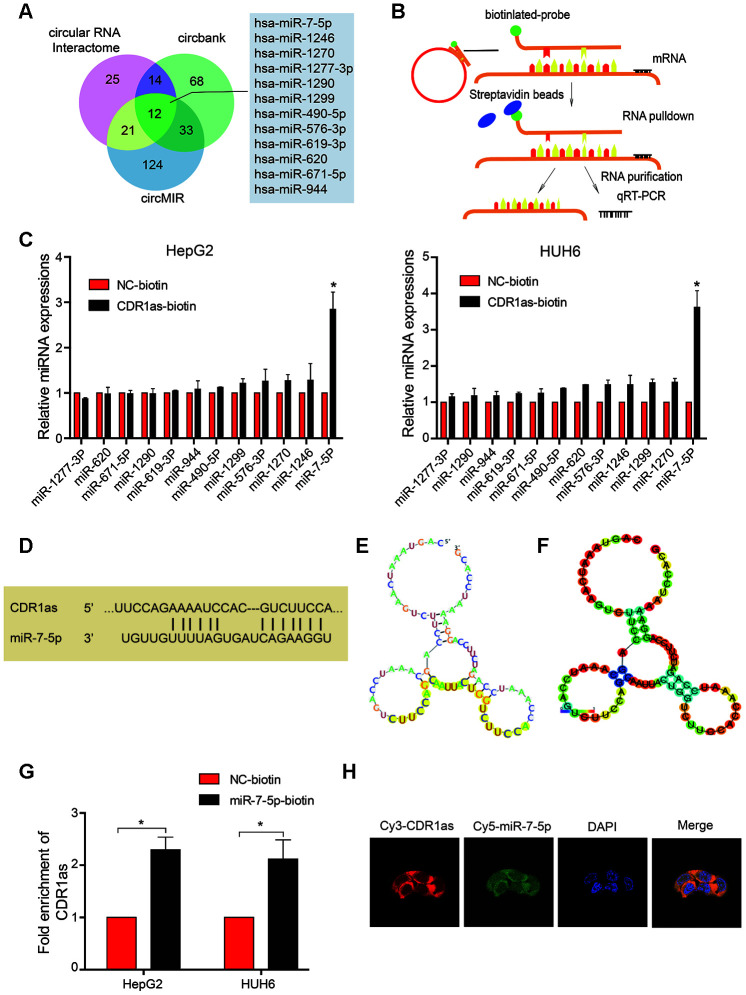
**CDR1as sponges miR-7-5p in HB cells.** (**A**) Schematic illustration showing overlapping target miRNAs of CDR1as that were predicted by the Circular RNA Interactome, Circbank, and circMIR; (**B**) Schematic diagram for the pull-down assay using biotinylated microRNA; (**C**) The relative level of 12 miRNA candidates in the HepG2 and HUH6 lysates was detected by real-time PCR. Multiple miRNAs were pulled down by CDR1as, and miR-7-5p was pulled down by CDR1as in two cell lines; (**D**) Potential binding sites between CDR1as and miR-7-5p; (**E**, **F**) The possible binding sites of CDR1as with miR-7-5p were predicted by RegRNA2.0, (**E**) Graph of predicted RNA secondary structure. The yellow region indicates the RNA fold predicted structure of the motif. (**F**) RNA fold reliability of pair probabilities; (**G**) The biotinylated wild-type miR-7-5p (miR-7-5p biotin) or its mutant (NC-biotin) were transfected into HepG2 and HUH6 cells. After streptavidin capture, CDR1as levels were quantified by real-time PCR. GAPDH was used as a negative control; (**H**) Fluorescence in situ hybridization (FISH) showed the colocalization between CDR1as and miR-7-5p. Data are presented as the mean ± SEM of three experiments. *P < 0.05 (Student’s t-test).

CircRNAs can act as miRNAs sponges that regulate downstream targets. In the current study, we observed that CDR1as was mainly located in the cytoplasm (Figure 1H–1I). Subsequently, to address whether CDR1as binds to miRNAs as a miRNA sponge in HB cells, we selected 12 candidate miRNAs by overlapping the results of the miRNA recognition elements in the CDR1as sequence that were predicted by Circular RNA Interactome, Circbank, and circMIR (Figure 3A). Next, we investigated whether CDR1as could directly bind to these candidate miRNAs. As shown in the Figure 3B, a biotin-labeled CDR1as probe was designed and verified to pull down CDR1as in the HB cell lines. The miRNAs were extracted after the pull-down assay, and the level of the 12 candidate miRNAs was detected by real-time PCR. As shown in Figure 3C, miR-7-5p was the most obvious miRNA that was abundantly pulled down by CDR1as in both the HepG2 and HUH6 cells. As predicted by the Circular RNA Interactome and RegRNA2.0, we found that miR-7-5p harbored seed sequence recognition sites that were complementary to CDR1as (Figure 3D–3F). Furthermore, we transfected biotinylated miR-7-5p mimics into HB cells, and the RNA captured by miR-7-5p was evaluated by qRT-PCR. Consistent with the results from the circRNA-miRNA pull-down assay, the biotinylated miR-7-5p mimics captured more CDR1as than the biotinylated control (Figure 3G). These findings demonstrated that miR-7-5p could also directly bind to CDR1as. Moreover, results from the FISH analysis in HB cells showed that CDR1as and miR-7-5p were co-localized in the cytoplasm (Figure 3H). Collectively, these results indicate that CDR1as can directly bind to miR-7-5p in the cytoplasm.

### MiR-7-5p inhibits proliferation and stemness of HB cells in vitro

**Figure 4:**
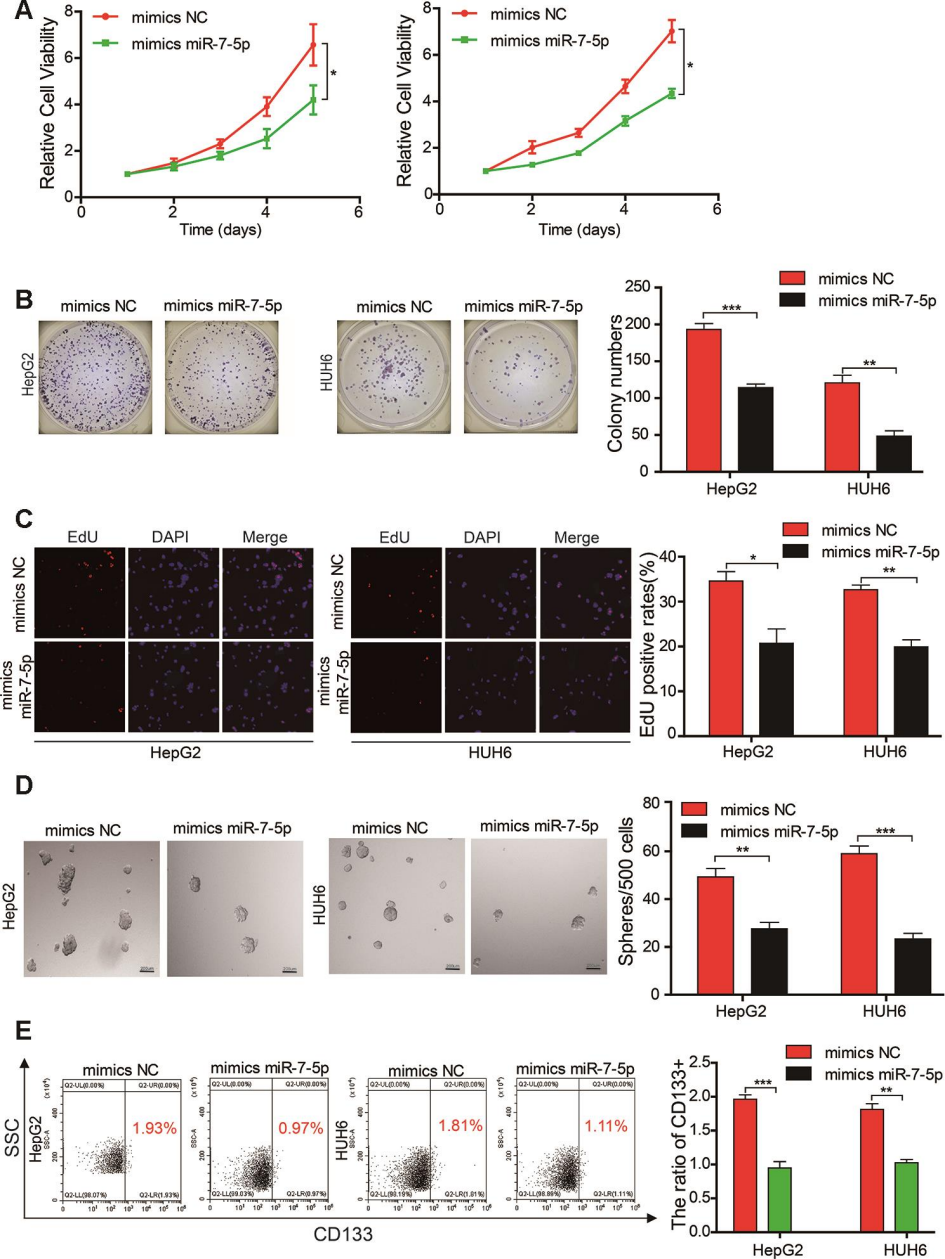
**Mir-7-5p affects the proliferation and stemness abilities of HB cells.** (**A**–**C**) The cell proliferative ability of HB cells that were transfected with miR-7-5p was assessed by the Cell Counting Kit-8 (CCK-8), colony formation assay, and EdU assay; (**D**) The stemness of cancer stem cells (CSCs) was assessed using the sphere-forming assay after the HepG2 and HUH6 cells were transfected with miR-7-5p; (**E**) The proportion of CD133+ cells was determined by flow cytometric analysis after miR-7-5p was overexpressed in HB cells. Scale bar, 200 μm. Data are presented as the mean ± SEM of three experiments. *P < 0.05,**P < 0.01,***P < 0.001 (Student’s t-test).

We transfected miR-7-5p mimics into HB cell lines, and results from the CCK-8 assay, colony formation experiment, and EdU assay indicated that the cell growth and colony-forming abilities of the HB cells that overexpressed miR-7-5p were inhibited as compared with those of the HB control cells (Figure 4A–4C). Results from the sphere-forming assay revealed that the sphere-forming capacity in the HepG2 and HUH6 cells that overexpressed miR-7-5p was significantly decreased as compared with that in the HB control cells (Figure 4D). The flow cytometric analysis revealed that the proportion of CD133+ cells was significantly decreased in the HB cells that overexpressed miR-7-5p as compared with that in the control cells (Figure 4E). These results revealed that miR-7-5p inhibited the proliferation and self-renewal ability of CSCs in the HB cells.

### MiR-7-5p affects the biological characteristics by targeting KLF4 in HB cells

**Figure 5:**
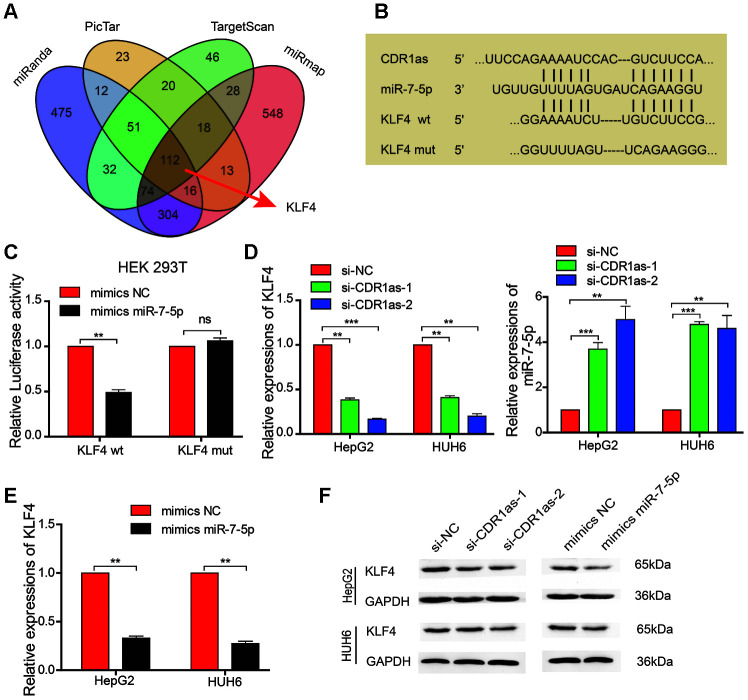
**MiR-7-5p affects the biological characteristics by targeting KLF4 in HB cells.** (**A**) Schematic illustration showing the overlapping possible target genes of miR-7-5p predicted by miRanda, PicTar, TargetScan, and miRmap; (**B**) Sequence alignment of CDR1as and KLF4 with miR-7-5p; (**C**) The relative luciferase activities were analyzed in HEK 293T cells that were co-transfected with miR-7-5p mimics or miR-mimics-NC and KLF4 wild type or Mut luciferase reporter vectors; (**D**) qRT-PCR analysis of the expression of KLF4 and miR-7-5p in HB cells that were treated with si-CDR1as; (**E**) qRT-PCR analysis of KLF4 mRNA in HB cells that were treated with miR-7-5p mimics; (**F**) Western blot analysis indicated that miR-7-5p downregulated KLF4, and this was similar to the effect of CDR1as siRNA on KLF4. Data are presented as the mean ± SEM of three experiments. **P < 0.01 (Student’s t-test).

We searched the miRanda, PicTar, TargetScan, and miRmap databases to identify possible downstream target genes of miR-7-5p in HB cells (Figure 5A). Although miR-7-5p was predicted to have approximately 112 downstream target genes, we discovered that KLF4 is associated with cancer stem cells and was ranked as a downstream target gene of miR-7-5p. We also obtained the binding sites between miR-7-5p and KLF4, and found that CDR1as and the 3′ UTR of KLF4 share the same miRNA response elements (MREs) as miR-7-5p. This suggests that there is a relationship between CDR1as and KLF4 (Figure 5B). To further confirm this relationship, we performed a dual-luciferase reporter assay and discovered that the luciferase activity was significantly reduced in HEK 293T cells that were co-transfected with miR-7-5p mimics and a luciferase reporter containing a wild-type 3′ UTR of KLF4 as compared with that in control HEK 293T cells. However, there was no difference in the luciferase activity between control HEK 293T cells and HEK 293T cells that were co-transfected with miR-7-5p mimics and a luciferase reporter that contained a KLF4 3′ UTR in which the predicted miR-7-5p binding site was mutated (Figure 5C). Results from qRT-PCR revealed that KLF4 expression was significantly decreased and miR-7-5p expression was significantly increased after CDR1as knockdown (Figure 5D). The qRT-PCR analysis showed that KLF4 expression was decreased after miR-7-5p upregulation (Figure 5E). The results from the Western blotting analysis revealed that the protein levels of KLF4 were decreased after the HB cells lines were transfected with si-CDR1as or miR-7-5p mimics (Figure 5F). These results revealed that miR-7-5p inhibited the proliferation and stemness of the HB cells by targeting KLF4.

### KLF4 knockdown inhibits proliferation and stemness of HB cells

**Figure 6:**
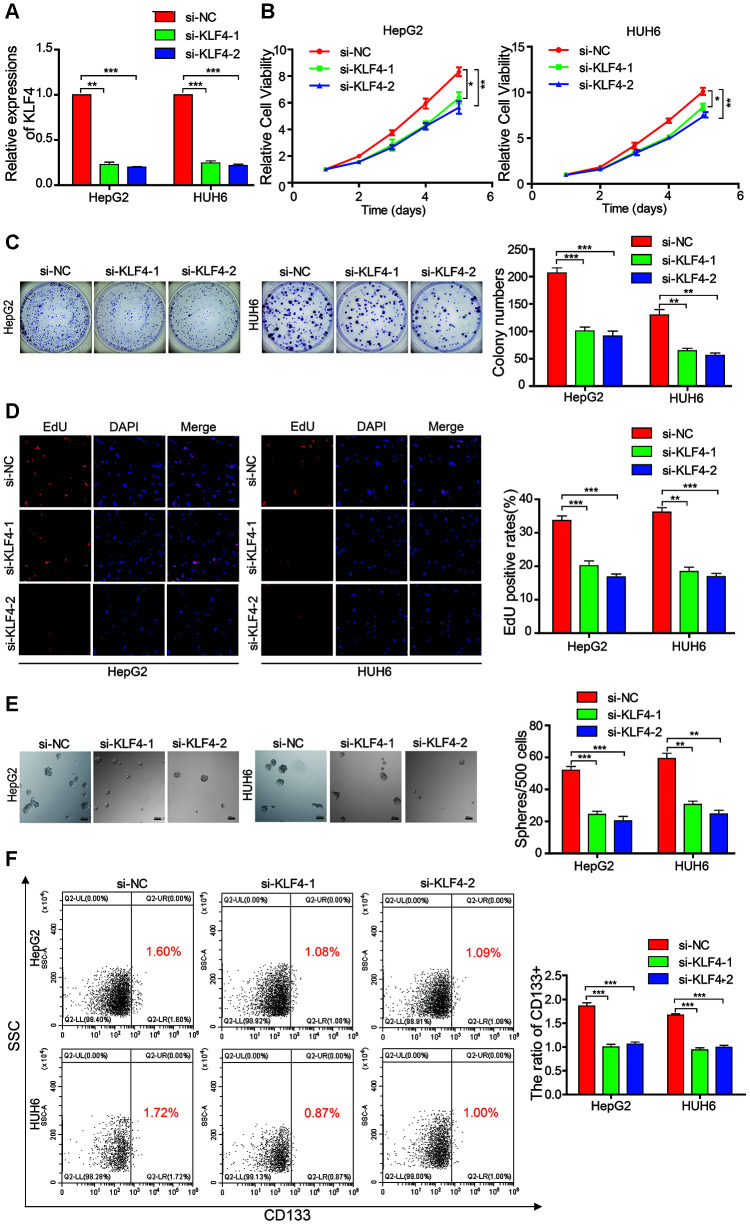
**KLF4 affects the proliferation and stemness abilities of HB cells.** (**A**) qRT-PCR analysis of KLF4 mRNA in HB cells that were treated with siRNAs; (**B**–**D**) The cell proliferative ability was assessed by the Cell Counting Kit-8 (CCK-8), colony formation assay, and EdU assay after KLF4 was knocked down in HepG2 and HUH6 cells; (**E**) The stemness of cancer stem cells (CSCs) was assessed by the sphere-forming assay after KLF4 was knocked down in HepG2 and HUH6 cells; (**F**) The proportion of CD133+ cells was determined by flow cytometric analysis after KLF4 knockdown. Scale bar, 200 μm. Data are presented as the mean ± SEM of three experiments. *P < 0.05,**P < 0.01,***P < 0.001 (Student’s t-test).

Although KLF4 acts as a oncogene in osteosarcoma, melanoma, and bladder cancer [18–20], the biological role of KLF4 in HB remains unknown. Therefore, we designed two siRNAs that targeted KLF4 to evaluate its influence on HB cells. Results from qRT-PCR showed that KLF4 successfully knocked down in the HB cells (Figure 6A). Results from the CCK-8 assay, colony formation experiment, and EdU assay indicated that the cell proliferation and colony-forming abilities of the KLF4 knockdown cells were inhibited as compared with those of HB control cells (Figure 6B–6D). The sphere-forming assay demonstrated that the sphere-forming capacity in the KLF4 knockdown cells was significantly decreased as compared with that in the HB control cells (Figure 6E). The flow cytometric analysis revealed that the proportion of CD133+ cells was significantly decreased in KLF4 knockdown cells as compared with that in the control cells (Figure 6F). These results revealed that the knockdown of KLF4 inhibited the proliferation and self-renewal ability of CSCs in the HB cells.

### miR-7-5p attenuates the function of CDR1as in HB cells

**Figure 7:**
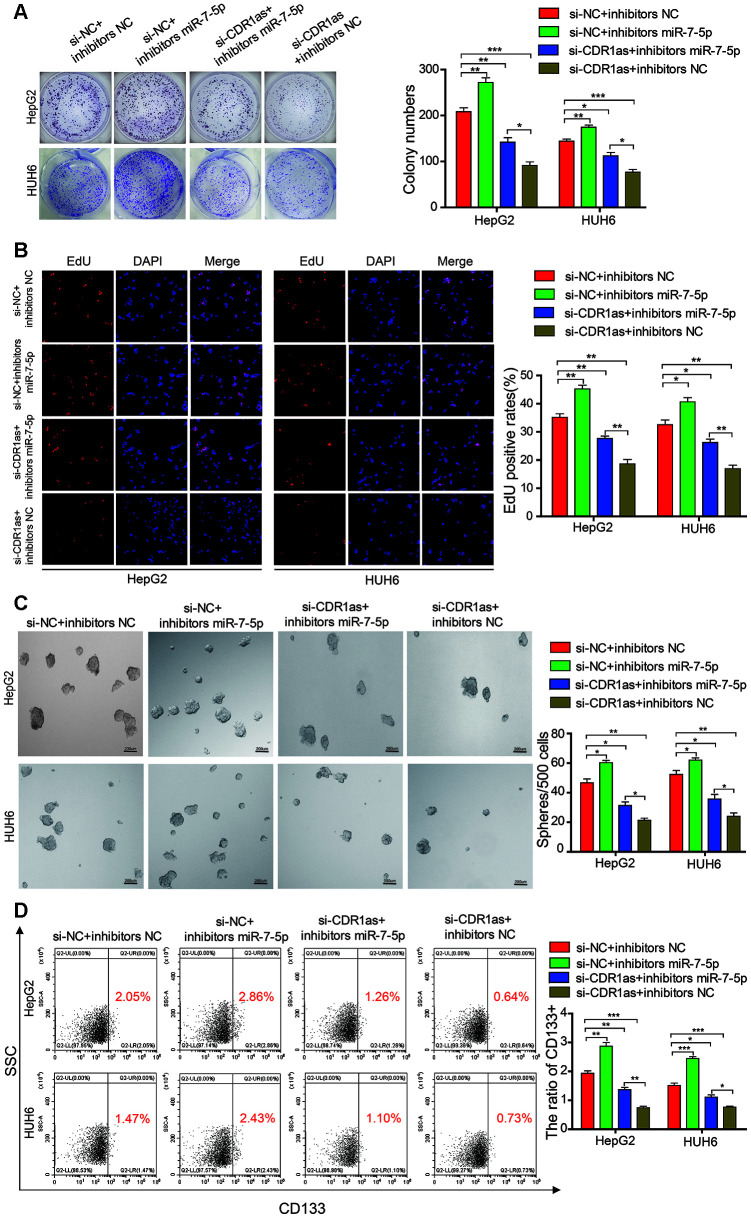
**MiR-7-5p attenuates the function of CDR1as in HB cells.** (**A**, **B**) The colony formation assay and EdU assay indicated that transfected with si-CDR1as could decrease the cell proliferative ability in HepG2 and HUH6 cells, and the co-transfection of si-CDR1as and miR-7-5p inhibitors abolished the si-CDR1as-induced attenuation of cell proliferation; (**C**) The sphere-forming assay indicated that transfected with si-CDR1as could decrease the sphere-forming capacity in HepG2 and HUH6 cells, and the co-transfection of si-CDR1as and miR-7-5p inhibitors abolished the si-CDR1as-induced attenuation of sphere-forming capacity; (**D**) The flow cytometric analysis indicated that transfected with si-CDR1as could decrease the proportion of CD133+ cells in HepG2 and HUH6 cells, and the co-transfection of si-CDR1as and miR-7-5p inhibitors abolished the si-CDR1as-induced decrease of CD133+ cells rate. Scale bar, 200 μm. Data are presented as the mean ± SEM of three experiments. *P < 0.05,**P < 0.01,***P < 0.001 (Student’s t-test).

Rescue experiments were performed by co-transfecting si-CDR1as (selected si-CDR1as-2) and miR-7-5p inhibitors in HB cells to determine whether CDR1as exerted a tumor-promoting effect on HB cells via sponging miR-7-5p. We found that the proliferative and colony formation abilities of HB cells that were co-transfected with miR-7-5p inhibitors and si-CDR1as were increased as compared with those of HB cells that were only transfected with si-CDR1as, suggesting that the knockdown of CDR1as partially abolished the miR-7-5p-induced suppression of proliferation (Figure 7A, 7B). The sphere-forming assay indicated that transfected with si-CDR1as could decrease the sphere-forming capacity in HepG2 and HUH6 cells, and the co-transfection of si-CDR1as and miR-7-5p inhibitors abolished the si-CDR1as-induced attenuation of sphere-forming capacity (Figure 7C). The flow cytometric analysis indicated that transfected with si-CDR1as could decrease the proportion of CD133+ cells in HepG2 and HUH6 cells, and the co-transfection of si-CDR1as and miR-7-5p inhibitors abolished the si-CDR1as-induced decrease of CD133+ cells rate (Figure 7D). Collectively, these results demonstrated that CDR1as inhibited the progression of the HB cells by impairing the anti-oncogenic role of miR-7-5p.

### CDR1as knockdown inhibits the growth of hepatoblastoma cells *in vivo*

**Figure 8:**
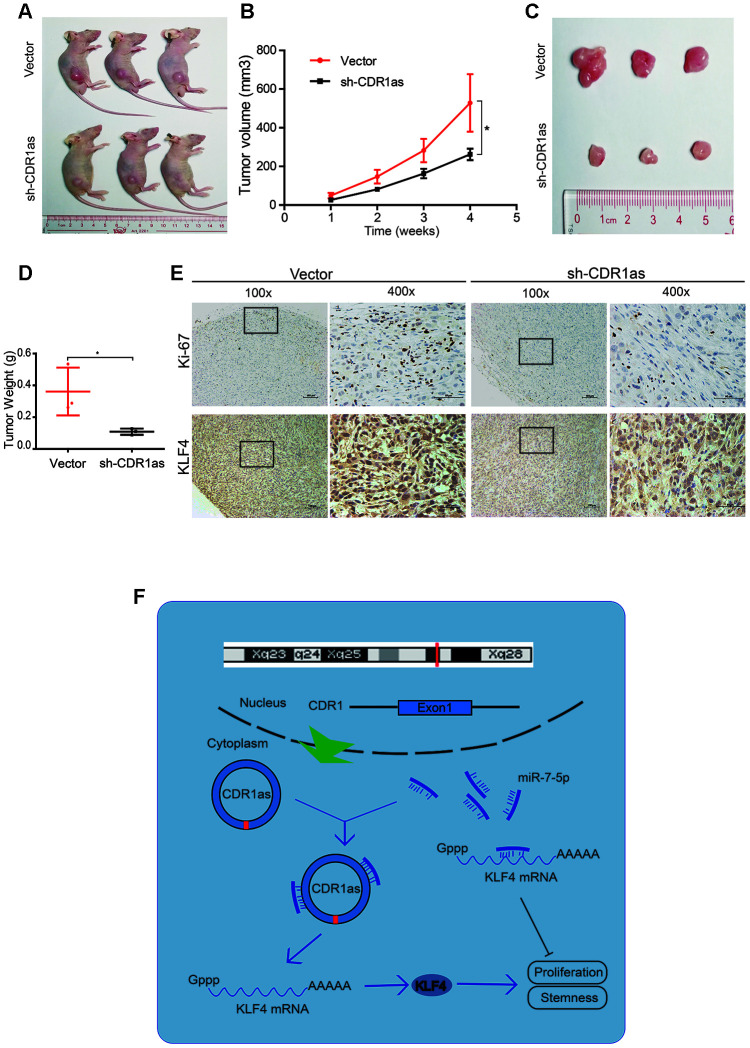
**CDR1as knockdown inhibits the growth of HB cells *in vivo.*** (**A**) Image of BALB/c nude mice that were subcutaneously injected with HepG2 cells (2 × 10^6^ cells per mouse; n = 3 per group); (**B**) The tumor volume of mice was measured weekly; (**C**) Image of subcutaneous xenograft tumors; (**D**) Tumor weights were significantly decreased in sh-CDR1as-treated mice; (**E**) Immunohistochemistry (IHC) of Ki-67 and KLF4 in the subcutaneous tumors; (**F**) Schematic illustration showing the relationship demonstrated in our study. Scale bar, 200 μm. Data are presented as the mean ± SEM of three experiments, *P < 0.05, **P < 0.01 (Student’s t-test).

To verify the effect of CDR1as on the regulation of HB growth in vivo, HepG2 cells that were stably transfected with a knocked down CDR1as lentivirus (sh-CDR1as) or a control lentivirus (vector) were subcutaneously injected into BALB/c nude mice. The tumor size was smaller and the tumor weight was lower in the sh-CDR1as group than the tumor size and weight in the control group (Figure 8A–8D). Immunohistochemical (IHC) analysis was applied to detect the protein expression of Ki-67, which is a marker for the proliferation of human tumor cells, and KLF4 in xenografted tumors of each group. The expression of Ki-67 and KLF4 was decreased in the tumor tissues of the sh-CDR1as group as compared with that in the control group (Figure 8E). Results from the qRT-PCR analysis also showed that the level of miR-7-5p was higher in the tumor tissues of the sh-CDR1as group as compared with that in the control group (Supplementary Figure 1). These results demonstrated that sh-CDR1as inhibited the growth of HB *in vivo* by regulating KLF4.

## DISCUSSION

Hepatoblastoma is a malignant embryonal tumor of the liver that consists of heterogenous populations of stem/progenitor cells [6–8]. Previous studies have demonstrated that CSCs may contribute to the origination and maintenance of cancers. CircRNAs are a novel class of RNA transcripts that are widely expressed in the mammalian genome; can function as potential diagnostic and prognostic biomarkers and as therapeutic targets in various diseases, including cancer; and may be involved in the regulation of CSCs. Previous studies have demonstrated that circRNAs play important roles in regulating the self-renewal of CSCs [12, 13]. However, there is currently no data on the expression of circRNAs in HB CSCs. Therefore, we explored how the expression of endogenous circRNAs affected the proliferation and differentiation of HB CSCs. We found that the circRNA, CDR1as, was highly expressed in CSC-enriched populations of HB cell lines, and the knockdown of CDR1as in the HB cell lines decreased the proportion of stem cells. These findings confirm that CDR1as plays a role in HB CSC maintenance. The knockdown of CDR1as also inhibited the proliferation and colony formation abilities of HB cells *in vitro*. These results suggest that CDR1as functions as an oncogene that promotes cancer stem cell-like characteristics in HB cells.

CircRNA can act as a sponge for miRNAs to regulate the expression of miRNA target genes in multiple human cancers, including hepatocellular carcinoma, renal cell carcinoma, and ovarian cancer [9–11]. We identified that CDR1as was located in cytoplasm (Figure 1H–1I), which indicates that CDR1as may regulate gene expression at the post-transcriptional level by sponging miRNAs. Then, we selected 12 candidate miRNAs by overlapping the miRNA recognition elements in the CDR1as sequence that were predicted by the Circular RNA Interactome, Circbank, and circMIR databases and verified that CDR1as interacted with miR-7-5p in HB cells using the biotinylated RNA pull-down and capture assays. We subsequently assessed the functional effects of miR-7-5p by transfecting miR-7-5p mimics into HB cells and found that miR-7-5p exerted an anti-oncogenic role on the HB cells. These results suggest that CDR1as may serve as an miRNA sponge for miR-7-5p.

Recent evidence indicates that circRNAs regulate gene expression by directly binding to miRNAs in order to prevent them from interacting with target genes. In our study, KLF4 was the predicted candidate target gene of miR-7-5p, and this was confirmed by the dual-luciferase reporter assay. Although KLF4 was initially defined as a tumor suppressor, the oncogenic role of KLF4 has become clearer in recent years. Specifically, KLF4 was recently classified as a critical initiator of early pancreatic cancer [33]. In addition, higher levels of KLF4 in breast cancer are usually associated with a high risk of tumorigenesis and a poor prognosis [34]. Therefore, a better understanding of the regulatory mechanisms of KLF4 may inhibit KLF4-associated tumorigenesis in HB. KLF4 is one of four key factors that is required for inducing pluripotent stem cells and is intimately implicated in the maintenance of the self-renewal capacity of embryonic stem cells. In addition, KLF4 maintains the stemness in osteosarcoma, breast cancer, and prostate cancer [18, 22, 23]. Our findings suggest that KLF4 promotes the proliferation of HB CSCs. Although the mechanisms underlying KLF4-regulated stemness in HB cells have not been completely elucidated, it is likely that its function as a direct transcriptional regulator plays a role in this process. A previous study also demonstrated that KLF4 activated the p38 MAPK signaling pathway to promote cancer stemness [18]. Additional evidence suggests that the KLF4-p21 axis plays an important role in maintaining the self-renewal and proliferation of CSCS [35, 36]. However, more research is needed to identify the role of KLF4 in CSCs.

Our study demonstrated that CDR1as was up-regulated in HB cell lines, tissues, and CSCs-enriched populations in HB cell lines. As shown in Figure 8F, the knockdown of CDR1as significantly inhibited the proliferation and stemness of HB cells by reducing the sponge activity on miR-7-5p and subsequently suppressing the interaction between miR-7-5p and KLF4. Results from this study suggest that CDR1as is an oncogene that influences HB stemness and implicates its application in HB therapy.

## MATERIALS AND METHODS

### Human cancer tissue and cell cultures

Human HB tissues were collected form patients who were undergoing resection of HB at the Sun Yat-sen Memorial Hospital (Guangzhou, China). Written informed consent was obtained from each patient or their guardians. This study was approved by the Institute Research Ethics Committee at this hospital.

HB cell lines, including HepG2 and HUH6 cells; a normal hepatocyte cell line, including LO2 cells; and HEK 293T cells were cultured in Dulbecco’s Modified Eagle’s medium (DMEM) supplemented with 10% fetal bovine serum (Bioind), streptomycin (100 μg/mL), and penicillin (100 μg/mL) (Gibco) and incubated at 37 °C in a humidified chamber with 5% CO2.

### RNA extraction and qRT-PCR

Total RNAs from the HB, adjacent normal liver tissues, and cells were extracted using the RNAiso Plus Kit (Takara, Japan) according to the manufacturer’s protocol. The RNA isolation of nuclear and cytoplasmic fractions was performed with NE-PER Nuclear and Cytoplasmic Extraction Reagents (Thermo Scientific, USA) according to the manufacturer’s protocol. Reverse transcription was performed with the Prime Script RT Master Mix (TaKaRa, Japan) for circRNA and mRNA. For miRNA, cDNA was initially synthesized using a miRNA First Strand cDNA Synthesis Kit (Sangon Biotech, China); then, it was subjected to real-time PCR in a Quantstudio™ DX instrument (Applied Biosystems, Singapore). GAPDH, small nuclear U6, and 18S rRNA were used as internal controls, and the 2-ΔΔCT method was used to calculate relative expression. The specific primers are listed in Supplementary Table 1.

### RNase R treatment

RNase R (Epicentre Technologies, USA) was used to degrade linear RNA. Briefly, RNAs were extracted from the HepG2 and HUH6 cells and split into two aliquots: 1) cells that underwent RNase R digestion and 2) cells in the control group (i.e., cells that were only treated with the digestion buffer). For RNase R digestion, 2 μg of total RNA was mixed with 0.6 μl 10 × RNase R Reaction Buffer and 0.2 μl RNase R (20 U/μl). For the control group, 2 μg of total RNA was mixed with 0.6 μl 10 × RNase R Reaction Buffer and 0.2 μl DEPC-treated water. Then, the samples were incubated at 37 °C for 30 min. GAPDH was used as an internal control in the control group. Three independent experiments were performed in triplicate.

### Oligonucleotide transfection

Small interfering RNAs (siRNAs), miRNA mimics, miRNA inhibitors, and negative control oligos were purchased from GenePharma (Shanghai, China). The sequences are listed in Supplementary Table 2. Cell transfection was conducted using the Lipofectamine RNAiMax Transfection Reagent (Life Technologies).

### Cell proliferation assay

The treated HepG2 and HUH6 cells were reseeded into 96-well plates (5 × 10^3^ cells per well), and cell proliferation was assessed using the CCK-8 assay (Beyotime). The absorbance of each well was measured at a wavelength of 450 nm using a SPARK 10 M spectrophotometer (Tecan, Austria).

To evaluate cell colony formation ability, the treated HepG2 and HUH6 cells were seeded into 6-wells plates (5 × 10^3^ cells per well) and incubated at 37°C in a 5% CO2 humidified atmosphere for 10 days. After incubation, the colonies were fixed with 4% paraformaldehyde, stained with 0.1% crystal violet, counted, and photographed.

### 5 -Ethynyl -2′ -deoxyuridine (EdU) assays

EdU assays were performed with the Cell-Light EdU DNA Cell Proliferation Kit (RiboBio, Guangzhou, China). Briefly, the treated cells (5 × 10^3^ per well) were seeded into each well of a 96-well plate and incubated for 48 h. Next, the cells were incubated with 100 ul of EdU for 2 h, fixed with 500 μl of 4% paraformaldehyde, and stained with Apollo Dye Solution. DAPI staining was used to identify the nucleic acid. Images of the cells were obtained with an inverted fluorescence microscope (Carl Zeiss, Jena, Germany), and the proportion of EdU-positive cells was calculated.

### Sphere formation assay

Single-cell suspensions were seeded at 500 cells per well in ultra-low attachment 96-well plates (Corning, Lowell, MA, USA) and cultured with DMEM/F12 supplemented with 20 ng/mL epidermal growth factor (R&D Systems), 20 ng/mL basic fibroblast growth factor (R&D Systems), and B-27 supplement (Gibco). The number and size of spheres that formed were evaluated under a microscope after 10 days.

### Flow cytometric analysis

HepG2 and HUH6 cells were harvested using trypsin-EDTA and washed 3 times with PBS. Next, 1 × 10^6^ cells were suspended in PBS and incubated with an anti-CD133 monoclonal antibody (Invitrogen) for 30 minutes at 4 °C in dark. After incubation, the cells were analyzed in a flow cytometer, and the isotype control was added for gating. The labeled cells were sorted by a flow cytometry into CD133+ or CD133− cells, and the data were collected and analyzed using the CytExpert_Setup flow cytometry system (Beckman Coulter, USA).

### Dual-luciferase assay

HEK 293T cells were seeded in 24-well plates at a density of 1 × 10^4^ cells per well for 24 h, co-transfected with the dual-luciferase reporter vector and miRNA mimics using the Lipofectamine RNAiMax Transfection Reagent (Life Technologies), and incubated for 48 h. After incubation, firefly and Renilla luciferase activities were measured using a dual-luciferase reporter assay system (Promega, USA) according to the manufacturer’s instructions. Relative luciferase activity was normalized to the firefly luciferase internal control. Independent experiments were performed in triplicate.

### Biotin-labeled probe pull-down assay

The biotin-labeled probe pull-down assay was performed as previously described [37, 38]. Briefly, the probe-coated beads were prepared by incubating the biotinylated CDR1as probe or oligo probe (RiboBio, Guangzhou, China) with Streptavidin-Dyna beads M-280 (Invitrogen, USA) at room temperature for 2 h. Then, approximately 1 × 10^7^ HB cells were fixed with 1% formaldehyde, lysed in lysis buffer, and incubated with the probe-coated beads at 4 °C overnight. The following day, the probe-Dyna bead-circRNA mixture was washed and incubated with 200 μl of the lysis buffer and proteinase K at room temperature for 2 h to reverse the formaldehyde crosslinking. Then, the RNA complexes were extracted using the RNAiso Plus Kit (TaKaRa, Japan) and detected by qRT-PCR. Three independent experiments were performed in triplicate.

### Biotin-labeled miRNA capture

HB cells were transfected with biotin-labeled miRNA mimics or nonsense controls (GenePharma, China) for 48 h. Streptavidin-Dyna beads M-280 were washed with lysis buffer and blocked with yeast tRNA on a rotator at 4 °C for 2 h. The cells were harvested, lysed, and incubated with the blocked beads at 4 °C overnight. The abundance of CDR1as in the bound fractions was determined by qRT-PCR. Three independent experiments were performed in triplicate.

### Fluorescence in situ hybridization (FISH)

The Cy3-labeled CDR1as probe was designed and synthesized by RiboBio (Guangzhou, China), and the Cy5-labeled miR-7-5p probe was designed and synthesized by GenePharma (Shanghai, China). The sequences of the probes are listed in Supplementary Table 3. The signals of the probes were detected using the Fluorescent In Situ Hybridization Kit (GenePharma, China) according to the manufacturer’s protocols. All images were acquired on a ZEISS LSM800 Confocal Microscope system (Carl Zeiss AG, Germany).

### Western blot analysis

Cells were lysed in RIPA buffer (CWBIO, China) with protease and phosphatase inhibitors (CWBIO, China). Next, identical quantities of proteins were electrophoresed by sodium dodecyl sulfate polyacrylamide gel electrophoresis (SDS-PAGE), transferred onto polyvinylidene fluoride PVDF membranes, and incubated with the following primary antibodies: KLF4 (1:1000 dilution, abcam, China) and GAPDH (1:5000 dilution, abcam, China) at 4 °C overnight. The following day, the membranes were incubated with appropriate horseradish peroxidase (HRP)-conjugated secondary antibodies at room temperature for 1 h. Signals were detected with the Immobilon ECL Substrate (Millipore, Germany) and visualized using the Immobilon™ Western Chemiluminescent HRP Substrate (Millipore, USA).

### Mouse model

All of the animal studies were approved by the Institutional Animal Care and Use Committee of the Institute of Biophysics, Chinese Academy of Sciences and conducted in compliance with its recommendations. After stable knockdown of CDR1as in 2×10^6^ HepG2 cells, they were subcutaneously injected into the back of 4-week-old male BALB/c mice. The tumor volume was monitored weekly for a total of 4 weeks. After 4 weeks, the mice were sacrificed and tumor tissues were excised and subjected to pathological examination.

### Immunohistochemical (IHC) analysis

Fresh tumor tissue samples from the nude mice were fixed in 4% paraformaldehyde, dehydrated in an ethanol solution, embedded in paraffin, and cut into 5 μm thick sections. The primary antibody for KLF4 (abcam, China) was used at a 1:1000 dilution in the experiments. Images were captured using a Nikon Eclipse 80i system with NIS-Elements software (Nikon, Japan).

### Statistical analysis

We performed our experiments in triplicate, and the results are presented as the mean ± standard deviation. All statistical analyses were performed using SPSS statistical software. The data were analyzed with Student’s t-tests, and p < 0.05 was considered statistically significant.

## Supplementary Material

Supplementary Figure 1

Supplementary Tables
