# Supplementary material for: CircRNA CDR1as promotes hepatoblastoma proliferation and stemness by acting as a miR-7-5p sponge to upregulate KLF4 expression
Source: Aging (Albany NY). 2020 Oct 14;12(19):19233–53. doi: 10.18632/aging.103748 (PMC7732296; doi:10.18632/aging.103748)
Supplement: Supplementary Figure 1 [file aging-12-103748-s001..pdf]

SUPPLEMENTARY FIGURE

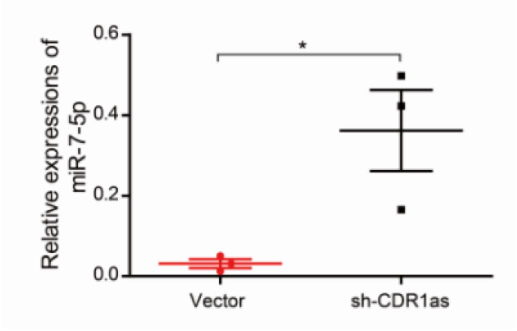

Supplementary Figure 1. qRT-PCR analysis of miR-7-5p in the tumor tissues of the sh-CDR1as group.
