# Supplementary material for: CircRNA CDR1as promotes hepatoblastoma proliferation and stemness by acting as a miR-7-5p sponge to upregulate KLF4 expression
Source: Aging (Albany NY). 2020 Oct 14;12(19):19233–53. doi: 10.18632/aging.103748 (PMC7732296; doi:10.18632/aging.103748)
Supplement: Supplementary Tables [file aging-12-103748-s002..pdf]

SUPPLEMENTARY TABLES

Supplementary Table 1. The primers used in this study are listed as follows.

Primers	Sequence (5'-3')
GAPDH Forward	GTCTCCTCTGACTTCAACAGCG
GAPDH Reverse	ACCACCCTGTTGCTGTAGCCAA
CircRNA CDR1as Forward	CAGTCTTCCATCAACTGGCTCA
CircRNA CDR1as Reverse	ACACAGGTGCCATCGGAAAC
CDR1 Forward	CGGATTTCCTGGAAGACCTGGA
CDR1 Reverse	TCCGTGTCTTCCAGCAAGTCCA
KLF4 Forward	CATCTCAAGGCACACCTGCGAA
KLF4 Reverse	TCGGTCGCATTTTTGGCACTGG
SOX2 Forward	GCTACAGCATGATGCAGGACCA
SOX2 Reverse	TCTGCGAGCTGGTCATGGAGTT
OCT4 Forward	CCTGAAGCAGAAGAGGATCACC
OCT4 Reverse	AAAGCGGCAGATGGTCGTTTGG
NANGO Forward	CTCCAACATCCTGAACCTCAGC
NANGO Reverse	CGTCACACCATTGCTATTCTTCG
CD133 Forward	CACTACCAAGGACAAGGCGTTC
CD133 Reverse	CAACGCCTCTTTGGTCTCCTTG
CD24 Forward	CACGCAGATTTATTCCAGTGAAAC
CD24 Reverse	GACCACGAAGAGACTGGCTGTT
CD13 Forward	GCTGTTTGACGCCATCTCCTAC
CD13 Reverse	GTTCTGGTAGGCAAAGGTGTGG
U6 Forward	CCGTATGACCTCCTTCCACAGA
U6 Reverse	TCTGTCCACCTCTGAAACCAGG
miR-7-5p Forward	ACACTCCAGCTGGGTGGAAGACTAGTAGTTTT
miR-7-5p Reverse	CTCAACTGGTGTCGTGGAGTCGGCAATTCAGTTGAGAACAACAA
miR-1246 Forward	ACACTCCAGCTGGGAATGGATTTTTGG
miR-1246 Reverse	CTCAACTGGTGTCGTGGAGTCGGCAATTCAGTTGAGCCTGCTCC
miR-1270 Forward	ACACTCCAGCTGGGCTGGAGATATGGAAGAG
miR-1270 Reverse	CTCAACTGGTGTCGTGGAGTCGGCAATTCAGTTGAGACACAGCT
miR-1277-3p Forward	ACACTCCAGCTGGGTACGTAGATATATATG
miR-1277-3p Reverse	CTCAACTGGTGTCGTGGAGTCGGCAATTCAGTTGAGAAAATACA
miR-1290 Forward	ACACTCCAGCTGGGTGGATTTTTGGAT
miR-1290 Reverse	CTCAACTGGTGTCGTGGAGTCGGCAATTCAGTTGAGTCCCTGAT
miR-1299 Forward	ACACTCCAGCTGGGTTCTGGAATTCTGTGT
miR-1299 Reverse	CTCAACTGGTGTCGTGGAGTCGGCAATTCAGTTGAGTCCCTCAC
miR-490-5p Forward	ACACTCCAGCTGGGCCATGGATCTCCAG
miR-490-5p Reverse	CTCAACTGGTGTCGTGGAGTCGGCAATTCAGTTGAGACCCACCT
miR-576-3p Forward	ACACTCCAGCTGGGAAGATGTGGAAAAATT
miR-576-3p Reverse	CTCAACTGGTGTCGTGGAGTCGGCAATTCAGTTGAGGATTCCAA
miR-619-3p Forward	ACACTCCAGCTGGGGACCTGGACATGTTTGTG
miR-619-3p Reverse	CTCAACTGGTGTCGTGGAGTCGGCAATTCAGTTGAGACTGGGCA
miR-620 Forward	ACACTCCAGCTGGGATGGAGATAGATAT
miR-620 Reverse	CTCAACTGGTGTCGTGGAGTCGGCAATTCAGTTGAGATTTCTAT
miR-671-5p Forward	CTCAACTGGTGTCGTGGAGTCGGCAATTCAGTTGAGCTCCAGCC
miR-671-5p Reverse	ACACTCCAGCTGGGAGGAAGCCCTGGAGGGG
miR-7-5p Forward	CTCAACTGGTGTCGTGGAGTCGGCAATTCAGTTGAGAACAACAA
miR-7-5p Reverse	ACACTCCAGCTGGGTGGAAGACTAGTGATTTT
miR-944 Forward	CTCAACTGGTGTCGTGGAGTCGGCAATTCAGTTGAGCTCATCCG
miR-944 Reverse	ACACTCCAGCTGGGAAATTATTGTACATCG
18S rRNA Forward	GGAGTATGGTTGCAAAGCTGA
18S rRNA Reverse	TCCTGCTTTGGGGTTCGATT

Supplementary Table 2. The oligonucleotides transfected in this study are listed as follows.

Oligonucleotides	Sequence (5'-3')
NC sense	UUCUCCGAACGUGUCACGUTT
NC antisense	ACGUGACACGUUCGGAGAATT
CDR1as si-1 sense	CUGCAAUAUCCAGGGUUUCTT
CDR1as si-1 antisense	GAAACCCUGGAUAUUGCAGTT
CDR1as si-2 sense	AUCCAGGGUUUCCGAUGGCTT
CDR1as si-2 antisense	GCCAUCGGAAACCCUGGAUTT
miR-7-5p mimics sense	UGGAAGACUAGUGAUUUUGUUGUU
miR-7-5p mimics antisense	CAACAAAAUCACUAGUCUUCCAUU
KLF4 si-1 sense	CCGAGGAGUUCAACGAUCUTT
KLF4 si-1 antisense	AGAUCGUUGAACUCCUCGGTT
KLF4 si-2 sense	CCUUACACAUGAAGAGGCATT
KLF4 si-2 antisense	UGCCUCUUCAUGUGUAAGGTT
MicroRNA inhibitor NC	CAGUACUUUUGUGUAGUACAA
miR-7-5p inhibitor	AACAACAAAAUCACUAGUCUUCCA

Supplementary Table 3. The probes used in this study are listed as follows.

	Sequence (5'-3')
**FISH Probes**	
Cy3-U6	TTTGCGTGTCATCCTTGCG
Cy3-18S	CTTCCTTGGATGTGGTAGCCGTTTC
Cy3-CDR1as	CCATCGGAAACCCTGGATAT
Cy5-miR-7-5p	AACAACAAAATCACTAGTCTTCCA
**Biotin-coupled probes**	
Biotin-NC	GGCACCGTACGTCAACTTAA
Biotin-CDR1as	CCATCGGAAACCCTGGATAT
Biotin-miR-7-5p	UGGAAGACUAGUGAUUUUGUUGUU
